# Genetic Isolation between the Western and Eastern Pacific Populations of Pronghorn Spiny Lobster *Panulirus penicillatus*

**DOI:** 10.1371/journal.pone.0029280

**Published:** 2011-12-15

**Authors:** Seinen Chow, Andrew Jeffs, Yoichi Miyake, Kooichi Konishi, Makoto Okazaki, Nobuaki Suzuki, Muhamad F. Abdullah, Hideyuki Imai, Toshie Wakabayasi, Mitsuo Sakai

**Affiliations:** 1 Stock Enhancement and Aquaculture Division, National Research Institute of Aquaculture, Yokosuka, Kanagawa, Japan; 2 Department of Marine Science, University of Auckland, Auckland, New Zealand; 3 Department of Marine Science, Nagasaki University, Nagasaki, Japan; 4 Research Center for Fisheries Oceanography and Marine Ecosystem, National Research Institute of Fisheries Science, Yokohama, Kanagawa, Japan; 5 Ishigaki Tropical Station, Seikai National Research Institute, Okinawa, Japan; 6 University of the Ryukyus, Okinawa, Japan; 7 Oceanic Resources Division, National Research Institute of Far Seas Fisheries, Yokohama, Kanagawa, Japan; University of Canterbury, New Zealand

## Abstract

The pronghorn spiny lobster, *Panulirus penicillatus*, is a circumtropical species which has the widest global distribution among all the species of spiny lobster, ranging throughout the entire Indo-Pacific region. Partial nucleotide sequences of mitochondrial DNA COI (1,142–1,207 bp) and 16S rDNA (535–546 bp) regions were determined for adult and phyllosoma larval samples collected from the Eastern Pacific (EP)(Galápagos Islands and its adjacent water), Central Pacific (CP)(Hawaii and Tuamotu) and the Western Pacific (WP)(Japan, Indonesia, Fiji, New Caledonia and Australia). Phylogenetic analyses revealed two distinct large clades corresponding to the geographic origin of samples (EP and CP+WP). No haplotype was shared between the two regional samples, and average nucleotide sequence divergence (Kimura's two parameter distance) between EP and CP+WP samples was 3.8±0.5% for COI and 1.0±0.4% for 16S rDNA, both of which were much larger than those within samples. The present results indicate that the Pacific population of the pronghorn spiny lobster is subdivided into two distinct populations (Eastern Pacific and Central to Western Pacific), with no gene flow between them. Although the pronghorn spiny lobster have long-lived teleplanic larvae, the vast expanse of Pacific Ocean with no islands and no shallow substrate which is known as the East Pacific Barrier appears to have isolated these two populations for a long time (c.a. 1MY).

## Introduction

Broad geographic distribution of marine benthic invertebrates may be invoked by dispersal of the pelagic larvae. Spiny lobsters of the genus *Panulirus* are confined to shallow-warm water habitat [1]. They occur along the continental coast and islands in tropical to warm-temperate regions of much of the world, where five species have been described from the Atlantic and more than 15 species from the Indo-Pacific Oceans [2]. The geographic distribution of individual species of *Panulirus* can be very wide presumably greatly aided by their phyllosoma larvae which are characterized by a very long pelagic life that can last from several months to more than a year in some species [3]. However, it is also true that some *Panulirus* spiny lobster species may have relatively narrow geographic distributions [2]. For example, the habitat of the Japanese spiny lobster *P. japonicus* is confined within a relatively small area of Northeast Asia. *Panulirus marginatus*, *P. brunneiflagellum* and *P. pascuensis* are endemic to Hawaii, Ogasawara and Easter Islands, respectively, while *P. interruptus* is distributed along a relatively narrow latitudinal range on the west coast of North America [2]. Investigations of the relationships between the distribution patterns of the spiny lobster species and ocean currents has invoked speculation that the long-lived teleplanic phyllosoma larvae can be transported over great distance from their natal origins and are capable of delaying metamorphosis until they encounter the specific physical and chemical cues similar to their home environments [4].

**Figure 1:**
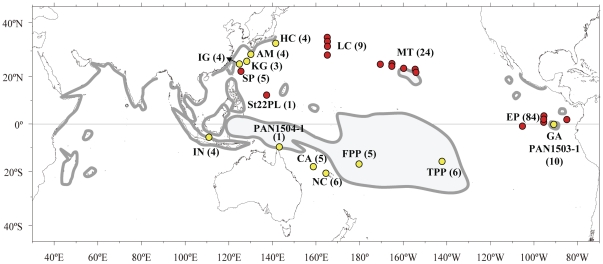
Collecting locations for the lobster samples used in this study are shown by yellow (adult) and red (larva) circles. Distribution of the pronghorn spiny lobster (*Panulirus penicillatus*) after Holthuis [2] is indicated by shaded line.

The pronghorn spiny lobster, *Panulirus penicillatus*, is a circumtropical species and characterized by probably the widest global distribution of any species of spiny lobster (Figure 1). Of all the spiny lobster species, only this species is known to occur in tropical and sub-tropical areas of both the eastern and western regions of the Pacific Ocean [2], [5], seemingly to have overcome the East Pacific Barrier (EPB) [6] which is generally considered as a significant ocean barrier for benthic invertebrates because it consists of a 4000–7000 km expanse of deep water without islands that separates the eastern Pacific (EP) from the central Pacific (CP). The long larval period of *P. penicillatus*, likely to last in excess of 8 months based on laboratory larval culture [7], may make larval transport across the EPB possible, but such a long larval period is common among *Panulirus* lobsters [3], [8], [9]. In January 2008, a large number of phyllosoma larvae of the genus *Panulirus* were captured during a research cruise by RV Kaiyo Maru (Fisheries Agency of Japan) operated in the Eastern Pacific. Two morphologically distinct types of phyllosoma were observed in the samples, one of which was identified to be *P. penicillatus*, since the larvae of this species at mid-to final stages of development are distinct in size and shape from those of the other congeneric species [7], [10]. We initially determined partial nucleotide sequence of the mtDNA cytochrome oxidase I (COI) of some of these Eastern Pacific phyllosoma larvae of *P. penicillatus*, and observed several nucleotides diagnostically different from those of the Western Pacific previously reported [10], [11]. Consequently, we collected and analyzed adults and phyllosoma larvae from other localities covering the entire Pacific Ocean to determine whether there was molecular genetic evidence that the Eastern Pacific pronghorn spiny lobster population is completely isolated from the Central and Western Pacific populations.

## Methods

### Ethics statement

All animal sampling in this study complied with the Council of the European Communities Directive 86/609/EEC regarding the protection of animals used for experimental and other scientific purposes, and fully complied with local fisheries management and marine protected area controls. Dead reptant lobsters were purchased from local commercial fishers and sampled and therefore no specific permits were required for the described field sampling as the fishers were required to comply with local laws regarding capture. Larval samples captured with plankton nets deployed from research vessels were dead on retrieval and sampled at this time, and all plankton net operations were carried out in high seas outside the Exclusive Economic Zone. Therefore the approval of coastal states was not required under the United Nations Convention on the Law of the Sea (UNCLOS). The species sampled are not endangered or protected.

### Lobster samples

**Figure 2:**
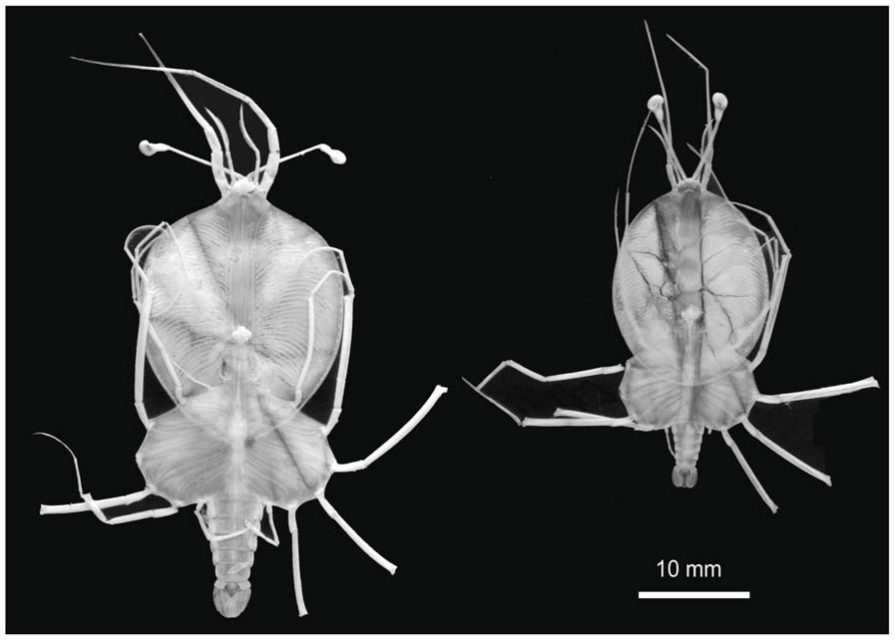
Two types of phyllosoma larvae of the genus *Panulirus* observed in the plankton samples collected in the western waters of Galápagos Islands. Both are final stage and the left one was morphologically identified to be *Panulirus penicillatus* but the right one could not be identified to a species.

Adults of *P. penicillatus* were collected from 11 locations (Table 1) and *Panulirus* phyllosoma larvae were collected from 17 locations (Table 2) from the Eastern, Central and Western Pacific Ocean (Figure 1). Adult lobsters were collected by local commercial fishers or personnel from research organizations, and the muscle tissues of pereiopod or abdomen were dissected on site, fixed in ethanol, and transferred to the laboratory. Ethanol preserved muscle tissues of one adult individual of *P. penicillatus* from Torres Strait (designated PAN1504-1) and Galápagos Is. (PAN1503-1) were kindly provided by Drs. M. J. Childress and M. B. Ptacek, Clemson University. Phyllosoma larvae of the genus *Panulirus* in the east and west of Galápagos Islands (1° S–4° N and 84°–96° W) of the Eastern Pacific (EP) were collected from the RV Kaiyo Maru, operated by the Fisheries Agency of Japan, in December 2008 to January 2009. Of two distinct morphs among 84 mid- to final stage *Panulirus* larvae, one type (N = 37) had morphological features entirely consistent with *P. penicillatus* (Figure 2, left) and the other type (N = 47) (Figure 2, right) could not be identified to species level but morphologically resembled previous descriptions of *P. gracilis* [12]. Among 24 *Panulirus* larvae collected from the RV Kaiyo Maru from the Central North Pacific (CP) in the north of Hawaiian Archipelago (25–30°N, 154–167°W) during January 2010, one (MT501) was morphologically determined to be *P. penicillatus* and the other could not be identified to species level but appeared to belong to phyllosoma species group 1 [13]. Nine larvae (LC105–111) collected from the RV Kaiyo Maru in the Western North Pacific (WP) during December 2009 to January 2010 were not *P. penicillatus* and were determined to belong to phyllosoma species group 1 by morphological characteristics. These larvae were caught using a mid-water trawl net (LC-100^2^-R3 net, mesh size 6 mm, Nichimo Co. Ltd., Japan) having a maximum mouth opening of 10×10 m. The LC net towing (2 to 3 knots) was operated near the surface (shallower than 15 m) at night (20∶00 to 3∶00). Five phyllosoma larvae (SP) of *P. penicillatus* were obtained from larval collections from a research cruise by the RV Shun-yo Maru, Fisheries Research Agency of Japan, south of Ryukyu Archipelago in November 2004 [10], [11]. One *P. penicillatus* larva (St22PL) was collected from the RV Shoyo Maru, operated by the Fisheries Agency of Japan, in the Philippine Sea in July 2010, using an Isaacs-Kidd midwater trawl net. The larvae were preserved in 70–99% ethanol on board and later transferred to the laboratory.

**Table 1:** Collection information of adult pronghorn lobster (*Panulirus penicillatus*) samples.

Area	Sample ID	Locality	Year	N
Eastern Pacific (EP)	PAN1503-1*	Galápagos Islands	1995	1
	GA	Galápagos Islands	2010	9
Central Pacific (CP)	TPP	Tuamotu	2010	6
Western Pacific (WP)	HC	Hachijo Islands, Japan	2009	4
	AM	Amami Islands, Japan	2008	4
	KG	Okinawa Islands, Japan	2008	3
	IG	Ishigaki Islands, Japan	2008	4
	IN	Java Sea, Indonesia	2008	4
	FPP	Fiji	2010	5
	NC	New Caledonia	2010	6
	CA	Chesterfield Islands	2010	5
	PAN1504-1*	Torres Strait, Australia	1996	1

*Muscle tissues were provided by Michael J. Childres and Margaret B. Ptacek.

**Table 2:** Collection information of lobster phyllosoma larvae of the genus *Panulirus*.

Area	Sample ID	Locality	Year	month	Nb/	Stage
Eastern Pacific (EP)	EPa	1°18′S, 106°05′W	2007	Nov	1 (0)	IX
	EPc	2°00′N, 84°59′W	2007	Dec	4 (0)	VIII–IX
	EPe	1°00′N, 96°00′W	2008	Jan	21 (14)	V–VIII
	EPf	2°00′N, 96°00′W	2008	Jan	55 (20)	VI–X
	EPh	4°00′N, 96°00′W	2008	Jan	3 (3)	IX–X
Central Pacific (CP)	MT312	28°59′N, 169°59′W	2010	Jan	10 (0)	VI–X
	MT409	29°59′N, 165°58′W	2010	Jan	5 (0)	VIII
	MT410-411	29°00′N, 165°59′W	2010	Jan	6 (0)	V–VIII
	MT501-8	27°05′N, 160°06′W	2010	Feb	1 (1)	IV
	MT614	26°59′N, 154°59′W	2010	Feb	1 (0)	VIII
	MT615	25°54′N, 155°00′W	2010	Feb	1 (0)	IX
Western Pacific (WP)	LC105	34°09′N, 164°59′E	2009	Dec	1 (0)	VI
	LC106	32°59′N, 165°02′E	2010	Jan	1 (0)	V
	LC108	30°59′N, 165°01′E	2010	Jan	5 (0)	V–VI
	LC111	27°57′N, 165°01′E	2010	Jan	2 (0)	VI
	SPa/	21–25°N, 123°–126°E	2004	Nov	5 (5)	V–X
	St22PL	12°12′N, 137°59′E	2010	Jul	1 (1)	VI

a/ Chow et al., (2006) [10].

b/ parenthesis indicates number of *Panulirus penicillatus* larvae morphologically determined.

### DNA analysis

A DNA extraction kit (GenomicPrep Cells and Tissue DNA Isolation Kit, Amersham Bioscience) was used for both adult muscle tissue and larval pereiopod samples. The procedures for the polymerase chain reaction (PCR), and amplification of the mitochondrial cytochrome oxidase I gene (COI) region followed by nucleotide sequence analysis are described elsewhere [11]. In the present study, two internal primers (COI369R and COI977F) were additionally used for sequence analysis, and the nucleotide sequences were 5′-GTGATGAAGTTAACGGCTCC-3′ and 5′-GACACCTACTACGTAGTAGC-3′, respectively. The sequences obtained were aligned using MEGA ver. 4.0 [14] followed by examination by eye. Neighbor-joining (NJ) tree based on Kimura's two-parameter distance (K2P) and maximum parsimony (MP) tree were constructed using MEGA ver. 4.0. Maximum likelihood (ML) analysis was adopted using PAUP* 4.0b10 [15] for exploring tree topologies and PhyML 3.0 [16] for bootstrap evaluation of the ML topology based on the optimal substitution model selected by Modeltest 3.06 [17].

Adult (N = 17) and larval (N = 3) *P. penicillatus* samples and morphologically unidentified larval specimens from EP, CP and WP were subjected to nucleotide sequence analysis of mitochondrial 16S rDNA using universal primers (16Sar-L and 16Sbr-H) [18]. Obtained sequences were subjected to database homology search. 16S rDNA sequences from *P. penicillatus* were aligned and subjected to phylogenetic analysis as mentioned above.

## Results

### COI sequence analysis in *Panulirus penicillatus*

**Figure 3:**
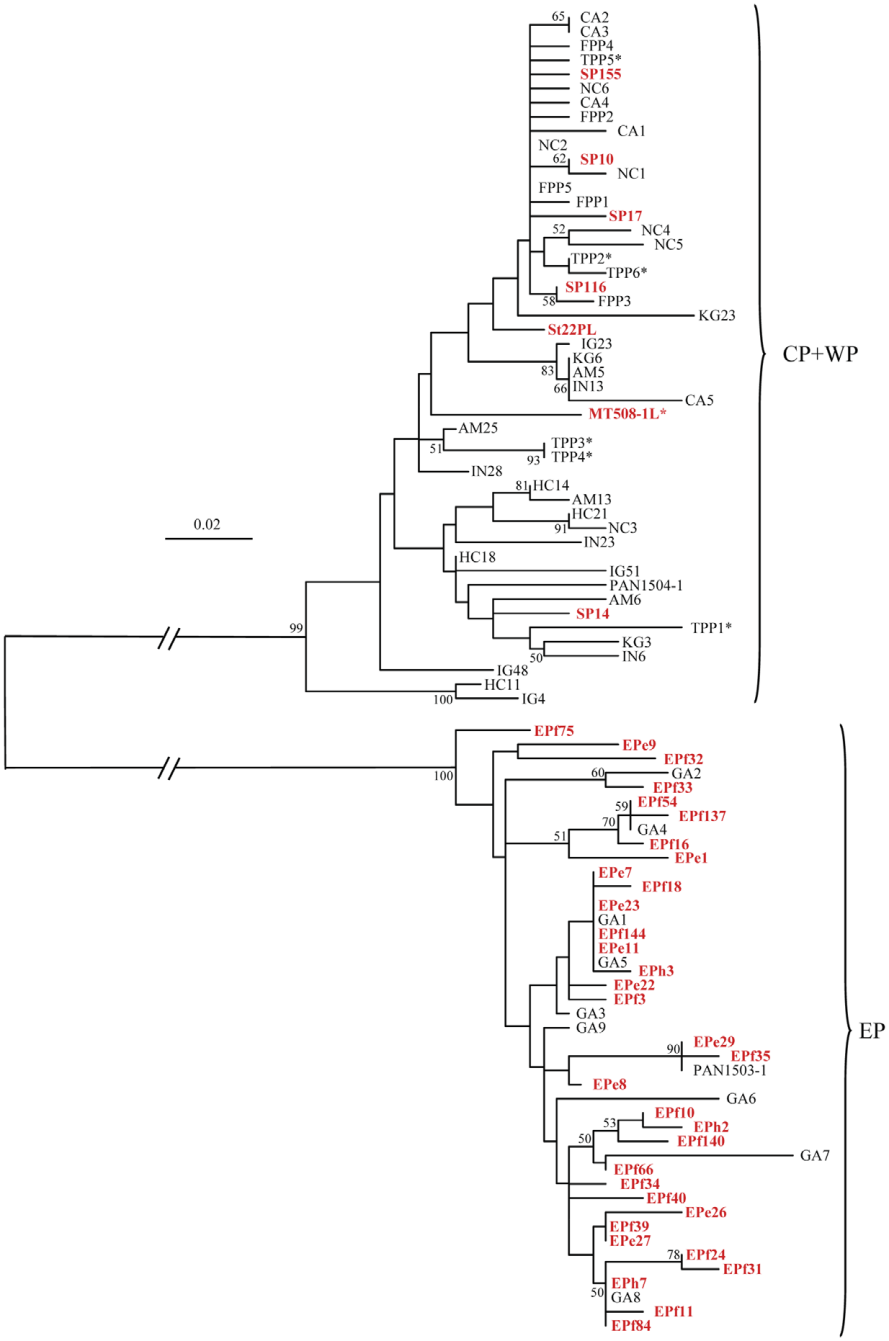
Neighbor-joining phylogenetic tree drawn using 1,103 bp COI sequences of 91 individuals of *Panulirus penicillatus* collected in the Eastern (EP), Central (CP) and Western Pacific (WP). Operational outgroup, *Panulirus japonicus* (NC_004251) [21] is not shown due to much longer branch than those within *P. penicillatus*. Bootstrap supports higher than 50% and numbers with branches indicate probability for 1,000 replications. Larval samples are shown in red. Samples carrying asterisk are from Central Pacific.

Partial nucleotide sequence (1,142–1,207 bp) of mtDNA COI region was determined for a total of 91 individuals of adult (N = 51) and larval (N = 40) *P. penicillatus*, with all initial morphological determinations of *P. penicillatus* larvae confirmed by the sequence data. All sequences are available in the database (DNA Data Bank of Japan: DDBJ accession numbers AB576698-AB576722, AB576726-AB576728, AB576730, AB576732-AB576747, AB576749-AB576755, AB610669-AB610707). Alignment of these sequences detected 76 haplotypes (Table S1). No indel was observed and 77 characters of 123 variable sites found in 1,103 bp were parsimony informative. All trees (NJ, MP and ML) rooted with *P. japonicus* COI sequence [19] were essentially the same in revealing two well-supported clades corresponding to the geographic origin of samples (EP vs. CP+WP)(Figure 3, only NJ tree is shown), to which 20 fixed nucleotide substitutions and 14 nearly fixed substitutions between EP and CP+WP samples appeared to be responsible (Table S1). Branching order within each clade varied among the trees and no phylogeographic assignment of individuals and no notable clustering of larval and adult samples within clade were observed. Thirty-two haplotypes were observed in 43 EP individuals and 44 haplotypes were observed in 48 CP+WP individuals, for which haplotype diversity in the entire sample was 0.993, and those within EP and CP+WP samples were 0.976 and 0.995, respectively. Average K2P distance between individuals of EP and CP+WP samples was 3.8±0.5%, and those between individuals within EP and CP+WP samples were 0.4±0.1% and 0.7±0.1%, respectively. Average K2P distance between *P. japonicus* and EP and CP+WP samples were 24.0±1.6% and 23.8±1.5%, respectively. The COI and 16S rDNA analyses indicated that the closest kin for *P. penicillatus* was an Atlantic species *P. echinatus* [20], both belonging to species group II [1]. Incorporation of the *P. echinatus* COI sequence (AF339454) [20] to our *P. penicillatus* data set could sample 573 bp. Average K2P distances between *P. echinatus* and total *P. penicillatus* sample was 12.4±1.5%, and between *P. echinatus* and EP and CP+WP samples were 12.1 ± 1.6% and 12.5±1.6%, respectively.

### 16S rDNA sequence analysis

**Figure 4:**
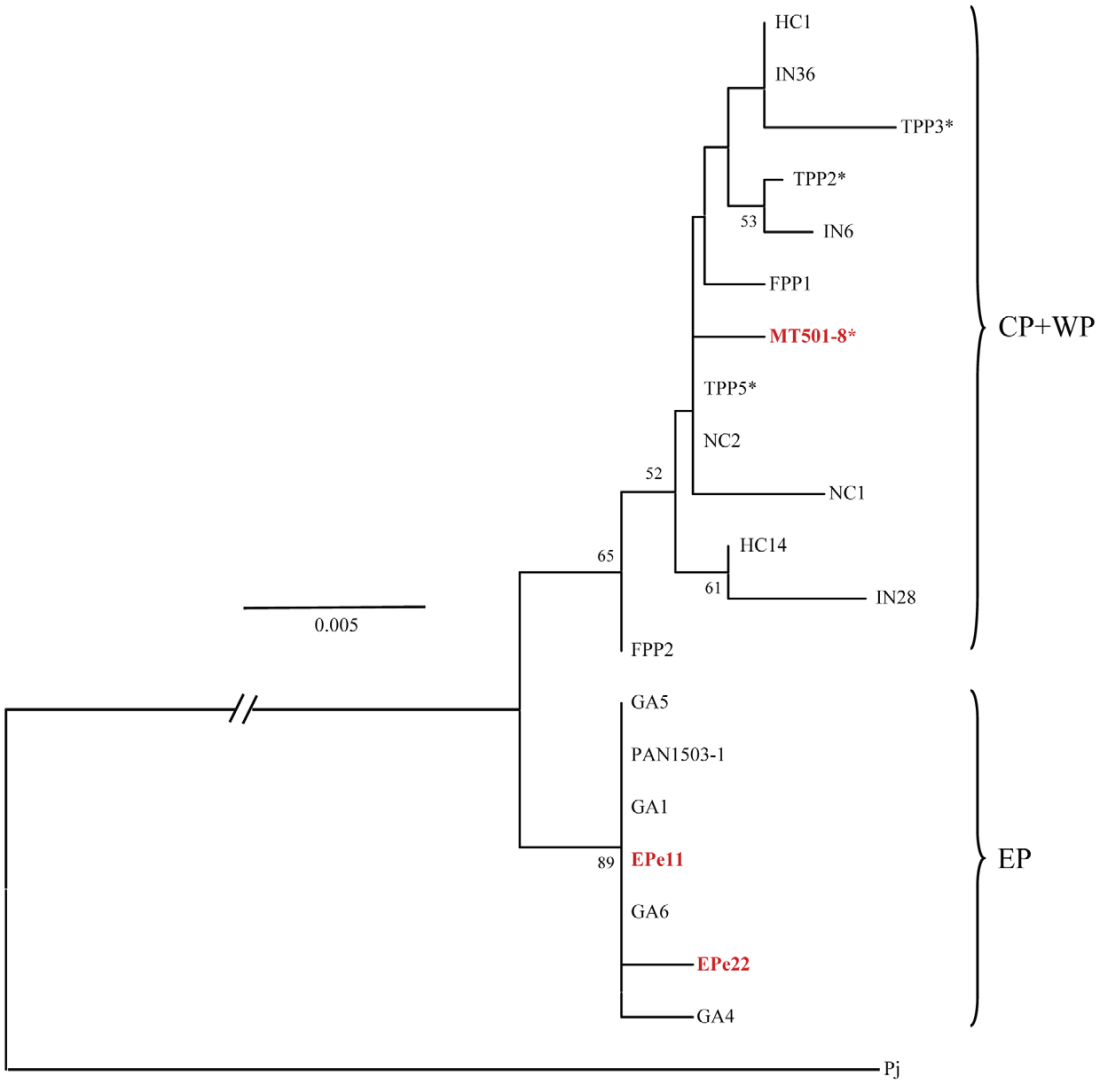
Neighbor-joining phylogenetic tree drawn using 536 bp 16S rDNA sequences of 20 individuals collected in the Eastern (EP), Central (CP) and Western Pacific (WP). The homologous sequence of *Panulirus japonicus* (NC_004251) [21] is used as an outgroup (Pj). Bootstrap supports higher than 50% and number with a branch indicates probability for 1,000 replications. Larval samples are shown in red. Samples carrying asterisk are from Central Pacific.

Partial 16S rDNA sequence (535 to 546 bp) was determined for two larvae and five adults from EP and for one larva and 12 adults from CP+WP samples of *P. penicillatus*, which were deposited to database (DDBJ accession numbers AB610708-AB610727). All *P. penicillatus* larvae initially sorted on the basis of morphology were confirmed with sequence data. Nucleotide sequence alignment for these twenty sequences detected 17 variable sites including one indel (Table 3). All trees (NJ, MP and ML) rooted with *P. japonicus* 16S rDNA sequence [19] agreed one another in that the tree topology is similar to those obtained using COI data (Figure 4, only NJ tree is shown). Total number of haplotypes was 14, and three and 11 haplotypes were observed in seven individuals from EP and 13 from CP+WP samples, respectively. Average K2P distance between individuals of EP and CP+WP samples was 1.0 ± 0.4%, and those within EP and CP+WP samples were 0.1±0.1% and 0.5±0.1%, respectively. Average K2P distances between the *P. echinatus* 16S rDNA (AF337965) [20] and all *P. penicillatus* samples was 4.6±1.0%, and those between *P. echinatus* and EP and CP+WP samples were 4.2±1.0% and 4.8±1.0%, respectively.

**Table 3:** Polymorphic nucleotide sites in 535 bp partial mitochondrial DNA 16S rRNA gene of 14 haplotypes detected in 20 individuals of *Panulirus penicillatus*.

	Nucleotide position	Number of individual							
Types	x2222222222223334	EP	GA*	MT	TPP	HC	IN	NC	FPP
	20344445578882694								
	83901573651692830								
1	TGTGGTATGGATG-TTT			1					
2	............A-...				1			1	
3	............A-..C					1			
4	............A-C..					1	1		
5	..........T.A-...				1				
6	..........T.A-C..						1		
7	.........A..A-...								1
8	..C.........A-...								1
9	.AA.........A-C..				1				
10	...A....T...A-...							1	
11	C....C......A-..C						1		
12	....A....A.CAT.C.	1	4						
13	....A..C.A.CAT.C.	1							
14	....A.G..A.CAT.C.		1						

*GA includes PAN1503-1.

The number of non-*P. penicillatus* larvae subjected to 16S rDNA analysis was 35 in EP, 23 in CP and nine in WP (Table 2), and all sequences were deposited to database (DDBJ accession numbers AB576663-AB576697, AB610728-AB610759). Homology search indicated that all 35 non-*P. penicillatus* larvae collected in EP matched to green spiny lobster (*P. gracilis*), all 23 non-*P. penicillatus* larvae collected in CP matched to the Hawaiian spiny lobster (*P. marginatus*), and nine non-*P. penicillatus* larvae in WP matched to the Japanese spiny lobster (*P. japonicus*).

## Discussion

### Genetic population structure and larval transport

It could be expected that there would be very little population structuring in *Panulirus* lobster species, as they all have long pelagic larval periods that typically last well over 6 months, and the larvae dwell in open ocean habitats where their distribution would be greatly influenced by physical ocean processes [21]. Indeed, homogeneous population structure is reported in several spiny lobster species [22], [23], [24]. However, spiny lobster species having very wide geographic distributions may show regional variation in population structure. For example, COI and 16S rDNA sequence analysis detected a large genetic difference between Brazilian and Western Atlantic/Caribbean populations of *P. argus*, sufficient to propose these two populations to be distinct species [25]. The large genetic difference between the *P. argus* populations was corroborated by mitochondrial DNA control region sequence analysis, in which two distinct subclades within the Western Atlantic/Caribbean sample were observed [26]. The present study revealed that there is no ongoing gene flow between the Eastern and Central-Western Pacific *P. penicillatus* populations. In contrast, very little evidence of population structuring was observed within each of these areas, a result which should be investigated further in more detail using larger sample sizes and more variable genetic markers. Despite the long pelagic larval period common to all spiny lobster species, a narrow distribution range is observed in several species of *Panulirus*. In an early review [4], larvae of spiny lobster species such as *P. japonicus*, *P. marginatus* and *P. interruptus*, endemic of northeast Asia, Hawaii and west coast of North America, respectively, were speculated to share the same ocean current system (the North Pacific Gyre) [27] but select different specific locations in which to settle. For example, *P. japonicus* larvae were predicted to return to Japan after four-years drifting in the North Pacific Gyre, whilst larvae of *P. marginatus* bypassed the coast of Japan using the same gyre to return back to Hawaiian waters. If these speculations were true, we would have observed phyllosoma larvae of different species in waters distant from their adult habitats. On the other hand, a much shorter route using the Kuroshio Current recirculation [27] was subsequently proposed for *P. japonicus* larvae [28], by which the larvae may return to Japan within a year, corresponding to the much shorter larval period of under a year observed in the laboratory culture of this species [8], [9]. The temporal distribution of the Japanese spiny lobster phyllosoma larvae in the southern waters of the Japanese Archipelago [10], [11] also supports the proposition of shorter route. A total of 310 phyllosoma larvae collected in the southern waters of the Japanese Archipelago were molecularly identified, but among this collection no *P. marginatus* was observed [10], [11], which might not be expected if larvae of this species were undertaking long distance transport on this current system to return to the Hawaiian Islands. In the present study, we also observed that phyllosoma species group 1 collected in the Northwest Pacific and Central North Pacific were exclusively *P. japonicus* and *P. marginatus*, respectively, contradicting the speculative long distance teleplanic dispersal scheme [4]. It appears that the larvae at least these two species of *Panulirus* may be entrapped by much narrower recirculation systems than previously postulated.

Four *Panulirus* species, *P. inflatus*, *P. interruptus*, *P. gracilis*, and *P. penicillatus*, are known from the tropical to temperate area of the Eastern Pacific [2]. Among them, *P. gracilis* has the widest distribution at the latitudinal range, being found along the continental coast from Baja California to northern Peru, including the Galápagos Islands. *Panulirus inflatus* and *P. interruptus* are confined to relatively higher latitude north of 15° and 20°, respectively. The main habitat of the Eastern Pacific *P. penicillatus* may be the Galápagos Islands [5], with a local fishery yielding nearly 100 tonnes in 1995 (frozen tail including *P. gracilis*) [29], and this species appears to be much less abundant on the neighboring continental coast to the east. Forty-four phyllosoma larvae collected outside the Gulf of California in November 2004 were molecularly identified [30], comprising 42 *P. inflatus* and 2 *P. gracilis*, but no *P. penicillatus* and *P. interruptus* were observed. Only *P. gracilis* larvae were observed in our phyllosoma sample collected east of the Galápagos Islands, and *P. penicillatus* together with *P. gracilis* larvae were observed west of Galápagos Islands. Thus, the pelagic distributions of these species of *Panulirus* phyllosoma broadly represent the distribution of the adult form on the adjacent continental shelf. Co-occurrence of *P. penicillatus* and *P. gracilis* larvae in our EP sample apparently indicate that these have drifted from Galápagos Islands and/or the coast of Central America. Larvae of both species were observed together in the same sampling cruises operated in regions further to the west (5° S to 15° N and 115° W to 125° W) [30], indicating that the *P. penicillatus* and *P. gracilis* larvae from the Eastern Pacific are penetrating into the middle of the East Pacific Barrier generally considered as a significant ocean barrier for benthic invertebrates because it consists of a 4000–7000 km expanse of deep water without islands [6]. *P. penicillatus* has previously been presumed to be unique among lobsters in having successfully overcome this barrier [31], but results of the present study have revealed that the population structure of *P. penicillatus* is consistent with the presence of this EPB with an absence of gene flow between the two populations on either side of the barrier. No larvae of the Western Pacific species such as *P. longipes*, *P. versicolor* and *P. ornatus* have been observed in the EPB, while larvae of Eastern Pacific lobsters have been found penetrating well into the EPB, suggesting that westward larval transport may be possible [31], especially in the vicinity of the equator [32]. However, the chances of larvae of the Eastern Pacific lobster species reaching oceanic islands in the Central Pacific have been expected to be low [31], and the countercurrents within the Equatorial Current System may provide routes for larvae that are taken westward to ultimately return to their natal coasts in the Eastern Pacific. Furthermore, diel vertical migration (DVM) behavior of phyllosoma larvae has been observed in many palinurid species and has been implicated in constraining the dispersal of these long-lived larvae, particularly when acting in tandem with retentive oceanographic processes [33], [34]. If this is also the case with *P. penicillatus* and *P. gracilis*, westward larval transport of these species from the EP may also be constrained by this DVM behavior. It may also be possible that whilst larvae do traverse the EPB they fail to successfully metamorphose due to an absence of the specific physical and/or chemical cues associated with their natal environment [4].

Regardless, the genetic evidence indicates that the larval supply, if any, from the eastern (Galápagos Islands) populations to the Central Pacific Islands may be extremely rare and insufficient to facilitate extensive gene flow across the EPB.

The EPB has been considered as a filter which allows long lived teleplanic larvae of invertebrates to pass whilst blocking the passage of others with shorter pelagic lives [35]. For example, the same mtDNA genotypes in a sea urchin species *Echinothrix diadema* was observed on both sides of the EPB, strongly suggesting that the populations of the Central and Eastern Pacific are connected by a high level of gene flow [36]. In this case, the pelagic larvae were expected to be transferred from the islands of the Central Pacific to those of the Eastern Pacific *via* the North Equatorial Counter-Current. The counter current was estimated to traverse the EPB for several months, and the transit time may be reduced to 50–81 days in a strong El Niño year [36]. Complete genetic isolation between Central and Eastern Pacific reef fish populations was observed only in two species among 20 examined, with both eastward and westward gene flow detected [37]. Rafting may resolve the paradox that palinurid lobsters with much longer pelagic larval periods cannot overcome the EPB while there is evidence that several invertebrate species and coral reef fishes can. Many species of coastal invertebrates and juvenile reef fish have been reported to associate with floating objects, including species of damselfish, corals, oysters, barnacles, polychaetes, bryozoans, tunicates, anemones [38], [39]. Therefore, long pelagic larval period may not be necessary for long-distance colonization of marine benthic invertebrates and coral reef fishes, if these species are capable of rafting for months. In contrast, the association of palinurid lobster larvae or early juveniles with flotsam has never been observed. Nevertheless, in order to finally determine whether the absence of gene flow across the EPB for *P. penicillatus* is due to physical oceanographic constraints, or behavioral constraints, such as an absence of metamorphosis cues, more extensive larval sampling and genetic analyses around the boundaries of the EPB will be necessary.

### Taxonomic status of the Eastern Pacific *Panulirus penicillatus* and its origin

The spiny lobster genus *Panulirus* is the largest group in the family Palinuridae, comprising 19 or more species [1], [2]. Morphological investigation and recent molecular analyses have revealed new species or sub-species within several extant species. Three sub-species in *P. homarus* complex ( =  *P. h. homarus*, *P. h. megasculpta* and *P. h. rubellus*) have been described based on the variation in coloration and abdominal transverse groove and carapace sculpturing [40]. A large genetic difference in mtDNA COI gene (14%, K2P distance) has been observed between *P. h. homarus* and *P. h. megasculpta* [20]. The *P. longipes* complex has also been morphologically broken down to several species and sub-species mainly on the basis of differences in coloration [2], [41]–[45], which are now well supported by molecular data [10], [11], [20], [25], [46]. Caribbean and Brazilian *P. argus* may be two different species or sub-species, in which nucleotide sequence difference in COI sequence was 25.8% (K2P distance) [25] comparable with, or even larger than, those between good species of *Panulirus* lobsters [20], [46]. According to the characteristic dark reddish-brown coloration of *P. penicillatus* in the Eastern Pacific [2], [5], [47], it has previously been indicated that this population was taxonomically distinct from its Central-Western Pacific counterpart [48], which has now been substantiated by the genetic results of the present study. The nucleotide sequence diversity in COI between the EP and CP+WP population of *P. penicillatus* was 3.8%, which is much smaller than that found between *P. homarus* sub-species [20] and between *P. argus* cryptic species [25], but comparable with that between *P. longipes longipes* and *P. l. bispinosus* [11], [46]. Furthermore, no haplotype was shared between EP and CP+WP populations of *P. penicillatus*. These indicate that the Eastern Pacific *P. penicillatus* may be ranked with cryptic sub-species and referred to as *P. penicillatus* ‘Red’ as postulated previously [48].

The closest kin for *P. penicillatus* is *P. echinatus* in the Atlantic, which is supported by both COI and 16S rDNA sequences [20] and morphologies of adult [1] and larvae [49]. A speculation that *P. echinatus* was initially widespread across the open Tethys Ocean and ultimately provided *P. penicillatus* as an isolated daughter species in the Pacific has been proposed [48]. In the genus *Panulirus*, two major lineages (I/II and III/IV) that correspond well to morphological species groups I + II and III + IV were observed by molecular analysis [20], while relationships between I and II and between III and IV within each major lineage were not resolved. However, all of their phylogenetic trees were in concordance in placing Atlantic and Eastern Pacific species including *P. penicillatus* at basal position in the I/II lineage. Therefore, the separation of *P. penicillatus* and *P. echinatus* from their common ancestor may be a relatively recent event corresponding to closing of the Isthmus of Panama, with subsequent immigration to Central and Western Pacific followed by complete isolation. We adopted 3.1MY for completion of the Isthmus of Panama [50], and K2P distance (12.4%) between *P. echinatus* and all *P. penicillatus* COI yielded evolutionary rates of 4% per MY. This value is similar to that (4.3% per MY) estimated for penaeid shrimp COI [51], but considerably larger than substitution rates estimated for several trans-isthmian species [52], [53]. Isthmian calibration may considerably vary according to the lineage selection, and considerable sources of error may be inherent in these estimates, including rate differences among taxa, [54]. Given that the substitution rate within a lineage may be constant, the K2P distance (3.8%) between EP and CP+WP populations of *P. penicillatus* suggests that successful trans-Pacific immigration from the Eastern Pacific was made 0.95MY ago and no gene flow has occurred since then.

## Supporting Information

Table S1 **Polymorphic nucleotide sites in 1,103 bp partial mitochondrial DNA COI gene of 76 haplotypes detected in 91 individuals of** ***Panulirus penicillatus*** **.**
(PDF)
