# Supplementary material for: Genetic Isolation between the Western and Eastern Pacific Populations of Pronghorn Spiny Lobster Panulirus penicillatus
Source: PLoS One. 2011 Dec 15;6(12):e29280. doi: 10.1371/journal.pone.0029280 (PMC3240654; doi:10.1371/journal.pone.0029280)
Supplement: Table S1 — Polymorphic nucleotide sites in 1,103 bp partial mitochondrial DNA COI gene of 76 haplotypes detected in 91 individuals of Panulirus penicillatus . (PDF) [file pone.0029280.s001.pdf]

Table S1. Polymorphic nucleotide sites in 1,103 bp partial mitochondrial DNA COI gene of 76 haplotypes detected in 91 individuals of *Panulirus penicillatus*.

Types	Nucleotide position
	111111111111222222222222222333333334444444444555555566666666667777777
	1234556788999012344577899000011344555669122233690123444589033477901444558990145688
	1087345703258706109869557136925928148069406725857324069284909826007147060681662125
1	AAATTACGATAGCGAGTGCTTGTCACAAAGAAACCTCAAGTGTGATTGAAACCTAGGCAAGAATATTTAGCGATATAAACAG
2	....T...........................
3	....C....................................
4	....C....................................
5	....C...............................G....
6	....C............G.......................
7	....C........G...........................
8	....C........G......................G....
9	....C........G......................G....
10	....C........G......................G....
11	....C........G......................G....
12	....C........G...................G..G....
13	....C........G..........C...........G....
14	....C........G.........G.................
15	....C........G......G...............G....
16	....C........G...G..................G....
17	....C........G..T...................G....
18	....C.....T..G......................G....
19	....C...G....G......................G....
20	....C..G.G..C.......................G....
21	....C.A......G......................G....
22	....C.A......T......................G....
23	....A...C......................G.............
24	......................................G......C....................................
25	....................................T.G......C....................................
26	..................................T........A......................................
27	...............................G......G.....GC...................................A
28	......................C......................C....................................
29	......................C......................C....................................
30	......................C......................C.........................A..........
31	......................C.G....................C....................................
32	.....................C.......................C....................................
33	....................C........................C........G......................G....
34	....................C........................C........G.....................GG....
35	...................C.........................C........GA.....................G....
36	..................A.........G........G.......C................G....C.........G....
37	..................G..................G.......C................G....C.........G....
38	.............A...............A...............C...G................................
39	...........A..........C......................C....................................
40	.........C...................................C.A.................................A
41	........G.......................G............C.......................AG......G....
42	........T....................................C.............G.................G..G.
43	.......A.....................................C........G...........................
44	.......A.....................................C........G...........C....A.....G....
45	..G.C.......TA..C...........C.G..T.C...A..A..C.........TA...C........A..G.G....A.A
46	.GG.C.......TA..C...........C.G..T.C...A..A..C.........TA...A........A..G.G...GA.A
47	.GG.C.......TA..C...........C.G..T.C...A..A..C.........TA...C........A..G.G...GA.A
48	.GG.C.......TA..C...........C.G..T.C...A..A..C.........TA...C........A..G.G...GA.A
49	.GG.C.......TA..C...........C.G..T.C...A..A..C.........TA...C........A..GCG...GA.A
50	.GG.C.......TA..C...........C.G..T.C...A..A..C.........TA...C........A..G.G...GA.A
51	.GG.C.......TA..C...........C.G..T.C...A..A..C.........TA...C........A..G.G....A.A
52	.GG.C.......TA..C...........C.G..T.C...A..A..C.........TA..GC........A..G.G...GA.A
53	.GG.C.......TA..C...........C.G..T.C...A..A..C......T..TA...C........A..G.G...GA.A
54	.GG.C.......TA..C...........C.G..T.C...AC.A..C.........TA...C........A..G.G...GA.A
55	.GG.C.......TA..C...........C.G..TTC...A..A..C.........TA...C........A..G.G...GA.A
56	.GG.C.......TA..C...........G.G..T.C...A..A..C.........TA..GC........A..G.G...GA.A
57	.GG.C.......TA..C..........TG.G..T.C...A..A..C.........TA...C........A..G.G...GA.A
58	.GG.C.......TA.AC...........C.G..T.C...A..A..C.........TA............A..G.G...GA.A
59	.GG.C.......TA.AC...........C.G..T.C...A..A..C.........TA...C........A..G.G...GA.A
60	.GG.C.......TA.AC...........C.G..T.C...A..A..C.........TA...C........A..G.....GA.A
61	.GG.C.......TA.AC...........C.G..T.C...A..A..C.........TA...C........A..G.G...GA.A
62	.GG.C.......TA.AC...........C.G..T.C...A..A..C........GTA...C........A..G.G...GA.A
63	.GG.C.......TA.AC...........C.G..T.C...A.AA..C......T..TA...C........A..G.G...GA.A
64	.GG.C.......TA.AC...........C.G..T.C...AC.A..C.........TA...C........A..G.....GA.A
65	.GG.C.......TA.AC...........C.G.GT.C...A.AA..C......T..TA...C........A..G.G...GA.A
66	.GG.C.......TA.AC......T....C.G..T.C...A..A..CC........TA...C........A..G.G...GA.A
67	.GG.C.....C.TA..C........AGTG.G..T.C...A..A..C.........TA...C........A..G.G...GA.A
68	.GG.C..A....TA..CA..........C.G..T.C...A..A..C.........TA...C........A..G.G...GA.A
69	.GG.C.T.....TA..C...........C.G..T.C...A..A..C......T..TA...C........A..G.G...GA.A
70	.GG.CG......T...C...........CAG..T.C...A..AA.C.........TA...C........A..G.G...GA.A
71	.GG.CG......TA..C...........C.G..T.C...A..A..C.........TA...C........A..G.G...GA.A
72	.GG.CG......TA..C...........CAG..T.C...A..A..C.........TA...C........A..G.G...GA.A
73	.GG.CG......TAG.C...........CAG..T.C...A..A..C.........TA...C........A..G.G...GA.A
74	.GG.CG....C.TA..C...........C.G..T.C...A..A..C.........TA...C..G.....A..G.GC..GA.A
75	.GGCC.......TA..C...........C.G..T.C...AC.A..C.........TA...C........A..G.G...GA.A
76	GGG.C.......TA..C...........C.G..T.C...A..A..C.........TA..GC........A..G.G...GA.A

Table S1. Continued

Types	Nucleotide position	Number of individual								
	111111111111111111<br>88888888888889999999999000000000000000001<br>02235677888991112445669001123344456677790<br>31704328147061470149582473924703684703673	EP	GA[a/]	MT	TPP	JP[b/]	SP[c/]	IN	NC[d/]	FPP
1	GACATTACAACACCAATATCCCTAAAAACCCCCGTGCAGGA					2		1		
2	....					1				
3	..........T........T......G............A.					1				
4	...G....G.T......................T.....A.				2					
5	...G......T............................A.					1				
6	..........T............................A.							1		
7	..........T.T............................				1					
8	..........T.T............................								1	1
9	..........T.T............G...............									1
10	..........T.T..G.........................								1	
11	...G......T.T.....C......................						1			
12	..........T.T............................									1
13	..........T..............................						1			
14	..........T.T............................				1					
15	..........T.T............................				1					
16	..........T.T............................									1
17	..........T.T............................						1			
18	..........T.T............................								1	
19	..........T.T............................								2	
20	..........T.T.............G..............					1				
21	..........T.T............................						1			
22	..........T.T............................									1
23	..T.......T........T..C...G............A.					1				
24	..........T........T..C...G........A...AG					1				
25	..........T..T.....T..C...G............A.							1		
26	A..								1	
27	..........T........T..C...G...........AA.				1					
28	.....C....T........T...G..G............A.					1				
29	....C.....T........T......G............A.					1				
30	.....C....T........T...G..G............A.								1	
31	....C.....T........T......G............A.					1				
32	..........T........T..C...G.T..........A.						1			
33	..........T.T............................						1			
34	..........T.T............................								1	
35	..........T.T............................								1	
36	......G...T........T.........T.........A.					1				
37	......G...T........T.........T.........A.					1				
38	..........T........T......G...........AA.								1	
39	..........T..........T....G...........CA.							1		
40	..........T........T......G..G.........A.					1				
41	..........T............................A.			1						
42	..........T........T...................A.					1				
43	..........T.T...........G................								1	
44	..........T.T............................								1	
45	...G..GTGGTG.G..C..TA.CC.......T....TG.A.	1								
46	...G..GTGGTG....C..TA.CC.......T....TG.A.		1							
47	...G..GTGGTG.G..C..TA.CC.......T....TG.A.	4	2							
48	...G..GTG.TG....C..TA.CC.G.....T....TG.A.	1								
49	...G..GTGGTG....C..TA.CC.......T....TG.A.	1								
50	A..G..GTGGTG.G..C..TA.CC.......T....TG.A.	1								
51	...G..GTGGTG.G..C..TA.CC.......T....TG.A.		1							
52	.G.G..GTGGTG....C..TA.CC.......T....TG.A.	1	1							
53	...G..GTGGTG....C..TA.CC.......T....TG.A.	2	1							
54	...G..GTGGTG....C..TA.CC.G.....TA...TG.A.	1	1							
55	...G..GTGGTG.G..C..TA.CC.......T....TG.A.	1								
56	.G.G..GTGGTG....C..TA.CC.......T....TG.A.	1								
57	......GTGGTG....C..TA.CC......AT..C.TG.A.		1							
58	...G..GTGGTG.G..C..TA.CC.......T....TG.A.	1								
59	...G..GTGGTG....C..TA.CC.......T....TG.A.	2								
60	...G..GTGGTG.G..C..TA.CC.......T....TG.A.	1								
61	......GTGGTG....C..TA.CC......AT..C.TG.A.	1								
62	...G..GTGGTG....A..TA.CC.......T....TG.A.	1								
63	...G..GTGGTG....C..TA.CC.......T....TG.A.	1								
64	A..G..GTGGTG.....G.TA.CC.......T....TG.A.	1								
65	...G..GTGGTG....C..TA.CC.......T....TG.A.	1								
66	...G..GTGGTG....C..TA.CC.......T....TG.A.	1								
67	...G..GTGGTG....C..TA.CC.......T....TG.A.		1							
68	...G..GTGGTG....C..TA.CC.......T....TG.A.	1								
69	...G..GTGGTG....C..TA.CC.......T....TG.A.	1								
70	...G..GTGGTG....C..TA.CC.......T....TG.A.	1								
71	...G..GTGGTG....C..TA.CC.......T....TG.A.	1								
72	...G..GTGGTG....C..TA.CC.......T....TG.A.	1								
73	...G..GTGGTG....C..TA.CC.......T....TG.A.	1								
74	...G..GTGGTG..G.C..TA.CC...G...T....TG.A.		1							
75	...G..GTGGTG....C..TA.CC.G.....TA...TG.A.	1								
76	.G.G..GTGGTG....C..TA.CC.......T....TG.A.	1								

[a/]GA includes PAN1503-1; [b/]JP includes HC, AM KG and IG; [c/]SP includes St22PL; [d/]NC includes CA.
